# Message Humanness as a Predictor of AI’s Perception as Human: Secondary Data Analysis of the HeartBot Study

**DOI:** 10.2196/67717

**Published:** 2026-02-03

**Authors:** Haruno Suzuki, Jingwen Zhang, Diane Dagyong Kim, Kenji Sagae, Holli A DeVon, Yoshimi Fukuoka

**Affiliations:** 1 Department of Physiological Nursing University of California, San Francisco San Francisco, CA United States; 2 Department of Communication University of California Davis, CA United States; 3 Department of Linguistics University of California, Davis Davis, CA United States; 4 School of Nursing University of California, Los Angeles Los Angeles, CA United States

**Keywords:** artificial intelligence, women, humanness, chatbot identity, anthropomorphism, human-AI interaction, AI, chatbots, healthcare, interaction, secondary analysis, messages, effectiveness, surveys, logistic regression, predictive, prediction, age, ethnicity, education, perceptions, conversations, health outcomes

## Abstract

**Background:**

Artificial intelligence (AI) chatbots have become prominent tools in health care to enhance health knowledge and promote healthy behaviors across diverse populations. However, factors influencing the perception of AI chatbots and human-AI interaction are largely unknown.

**Objective:**

This study aimed to identify interaction characteristics associated with the perception of an AI chatbot identity as a human versus an artificial agent, adjusting for sociodemographic status and previous chatbot use in a diverse sample of women.

**Methods:**

This study was a secondary analysis of data from the HeartBot trial in women aged 25 years or older who were recruited through social media from October 2023 to January 2024. The original goal of the HeartBot trial was to evaluate the change in awareness and knowledge of heart attack after interacting with a fully automated AI HeartBot chatbot. All participants interacted with HeartBot once. At the beginning of the conversation, the chatbot introduced itself as HeartBot. However, it did not explicitly indicate that participants would be interacting with an AI system. The perceived chatbot identity (human vs artificial agent), conversation length with HeartBot, message humanness, message effectiveness, and attitude toward AI were measured at the postchatbot survey. Multivariable logistic regression was conducted to explore factors predicting women’s perception of a chatbot’s identity as a human, adjusting for age, race or ethnicity, education, previous AI chatbot use, message humanness, message effectiveness, and attitude toward AI.

**Results:**

Among 92 women (mean age 45.9, SD 11.9; range 26-70 y), the chatbot identity was correctly identified by two-thirds (n=61, 66%) of the sample, while one-third (n=31, 34%) misidentified the chatbot as a human. Over half (n=53, 58%) had previous AI chatbot experience. On average, participants interacted with the HeartBot for 13.0 (SD 7.8) minutes and entered 82.5 (SD 61.9) words. In multivariable analysis, only message humanness was significantly associated with the perception of chatbot identity as a human compared with an artificial agent (adjusted odds ratio 2.37, 95% CI 1.26-4.48; *P*=.007).

**Conclusions:**

To the best of our knowledge, this is the first study to explicitly ask participants whether they perceive an interaction as human or from a chatbot (HeartBot) in the health care field. This study’s findings (role and importance of message humanness) provide new insights into designing chatbots. However, the current evidence remains preliminary. Future research is warranted to understand the relationship between chatbot identity, message humanness, and health outcomes in a larger-scale study.

## Introduction

Artificial intelligence (AI) chatbots are computer programs using natural language processing, machine learning, and large language models to simulate human-like conversations [1]. The advantages of using AI chatbots in health care include 24×7 availability, cost-effectiveness, and scalability. In contrast, incorrect responses, misleading advice, lack of empathy, or nuanced communication are often concerns of AI chatbot use. Given the recent rapid development of large language models, the application of AI chatbots in health care has been widely investigated. Recently, several systematic reviews and meta-analyses have examined the efficacy of AI chatbots in preventing or managing chronic illnesses. To summarize, AI chatbot–based programs have shown promising results in improving mental health [2-4], such as depressive or anxiety symptoms, diabetes management [5], promoting healthy diets [6], and increasing cancer screenings [7]. Moreover, our research team developed the AI chatbot behavior change model [8] and then initiated an AI chatbot development project (hereafter called HeartBot) aimed at increasing women’s knowledge and awareness of heart attacks in the United States. Recently, we published the promising results of these HeartBot trials [9,10].

Assessing whether participants perceive an interaction as human or from a chatbot is important because a perceived human interaction tends to increase trust, engagement, satisfaction, and expectancy effects [11-13]. Several well-designed, high-quality randomized controlled trials (RCTs) of chatbot interventions in health care have been conducted. However, these RCTs of health chatbots focused on measuring health outcomes [14-18], and in some studies, related constructs such as engagement and usability. None of these RCTs used direct perception questions for the participants, such as “Did you think you were texting to a human or a chatbot?” (or an equivalent direct perception question). Thus, strong evidence is still lacking to directly quantify the impact of health outcomes depending on whether the participants perceived humans or chatbots for the intervention.

To address this knowledge gap, we conducted a secondary data analysis of the HeartBot trial to examine how participants perceived HeartBot identity as a human versus an AI chatbot and to explore factors associated with perceptions of chatbot identity. In the HeartBot trial, the chatbot introduced itself as HeartBot at the beginning of the conversation, but it did not explicitly indicate that participants would be interacting with an AI system. To the best of our knowledge, this is the first study to explicitly ask participants whether they perceive an interaction as human or from a chatbot (HeartBot) in the health care field. Thus, the findings of the proposed secondary analysis can provide unique, preliminary evidence for future health care research.

## Methods

### Design and Sample

We conducted a secondary analysis of the HeartBot trial, a quasi-experimental study. Study details have been reported in studies by Fukuoka et al [9] and Kim et al [10]. In brief, the HeartBot trial aimed to evaluate the usability and potential efficacy of the fully automated AI HeartBot in increasing women’s awareness and knowledge of heart attack risk and symptoms. Eligible participants were invited to interact with the HeartBot through SMS text messaging. The eligibility criteria included women aged 25 years or older, residing in the United States, proficient in English, possessing a cell phone with texting capabilities, having internet access, without self-reported cognitive impairment or a history of heart disease or stroke, and not being a health care provider or student in a health care–related field. We followed the STROBE (Strengthening the Reporting of Observational studies in Epidemiology) reporting guidelines [19] (Multimedia Appendix 1).

### Conceptual Framework for HeartBot

When we began the HeartBot project, our research team could not identify a suitable conceptual framework for the project. Thus, we conducted a literature review and developed a new conceptual framework, the AI chatbot behavior change model, to guide the design and evaluation of chatbots for health behavior change. The detailed description of this framework was published in 2020 [8], and since then, it has been cited in other published studies. In brief, the AI chatbot behavior change model consists of four major domains: (1) designing the chatbot characteristics and understanding user backgrounds, (2) building relational capacity, (3) building persuasive conversational capacity, and (4) evaluating mechanisms and outcomes. Multimedia Appendix 2 provides explanations for each domain, along with relevant examples. The proposed secondary analysis is explored in domain 4, “evaluating mechanisms and outcomes,” including conversational quality (eg, message humanness) and user experiences (eg, message effectiveness). We acknowledge that we cannot thoroughly examine all subdomains listed in domain 4, since this study is a secondary analysis of the HeartBot trial. However, the preliminary findings from this study can help further improve the AI chatbot behavior change model and may ultimately assist in designing and evaluating AI chatbots in health care more effectively.

### HeartBot Intervention

Details of HeartBot were published in studies by Fukuoka et al [9] and Kim et al [10]. HeartBot was developed by investigators using the Google Dialogflow CX platform [20], a natural language understanding platform to create virtual agents. HeartBot connected with Twilio [21] for inputs from participants and output from HeartBot to be sent to each other over SMS text messages. Messages for HeartBot were manually crafted, including the potential responses. HeartBot conversed about topics such as symptoms, risk factors, and treatment of heart attacks, and the investigators checked the readability of HeartBot messages. The content of HeartBot was developed and tested by the cardiovascular experts and investigators based on the latest guidelines and evidence to ensure full control over the content presented to participants and to minimize the risk of having the system dispense false or misleading information. At the beginning of the conversation, the chatbot introduced itself as HeartBot; however, it did not explicitly indicate that participants would be interacting with an AI system. In addition, personalization and empathic responses were included to improve participants’ experience and engagement. For participants’ safety, the introduction message included the following medical emergency notice: “If you are experiencing a medical emergency, please call 911 immediately.”

### Procedure

Participants were recruited through social media (eg, Meta’s Facebook and Instagram) advertisements placed from October 2023 to January 2024, using targeting strategies that aim to reach racially and ethnically diverse demographics (eg, Hispanic or Latino and Black or African American women). Those interested in the research were redirected to an online screening form, which included the study aims, procedures, and benefits and risks of participation. The research team contacted the potential participants who met all eligibility criteria and asked them to sign an electronic consent form. Upon obtaining written consent, participants were asked to complete an online baseline survey consisting of sociodemographic status, cardiovascular risks, medication intake, and previous AI chatbot use. After confirming the completeness of the online survey, the research staff provided the study telephone number to start the conversation with HeartBot, where they could exchange SMS text messages with HeartBot. The participants were able to interact with HeartBot 24 hours a day, 7 days a week, from anywhere in the United States. Research staff monitored the conversations between HeartBot and participants to ensure participants’ safety and verify the accuracy of information provided by HeartBot. After 4 to 6 weeks of the HeartBot interaction, participants were asked to complete an online postintervention survey, including AI chatbot interaction experience and evaluations. All online surveys were administered by Research Electronic Data Capture (Research Electronic Data Capture) [22], a secure online tool used to manage study data.

### Measures

#### Baseline Measures: Sociodemographic Characteristics, Cardiovascular Risks, Medication, and Past AI Chatbot Use

Sociodemographic factors, such as age, race or ethnicity, education, household income, marital status, employment status, and immigration experience to the United States, were collected from participants in the baseline survey. Data collected at baseline included self-reported cardiovascular risks, including menopause, BMI (kg/m^2^; calculated with height and weight), smoking in the past 30 days, physical activity ≥150 mins per week, family history of heart disease, prescribed blood pressure, cholesterol, diabetes medication, and daily aspirin intake. The cardiovascular risk factor variables were selected based on the latest clinical guidelines [23]. We assessed past AI chatbot use experience with the following question: “*Have you used any chatbot in the past 30 days*?” There were 2 response options—yes and no.

#### Postintervention Measures

##### AI Chatbot Interaction

In the postintervention survey, we measured several metrics indicating users’ interaction patterns with HeartBot, including users’ word count, the time spent in conversation in minutes, and the number of questions asked by users.

##### Message Humanness

In the AI chatbot behavior change model [8], message humanness is categorized as the “conversational quality” in domain 4, “evaluating mechanisms and outcomes,” which measures the degree of perceived humanness in chatbot conversations. Participants rated the humanness of the message using the “anthropomorphism scale” [24] in the postintervention survey. The scale consists of 5 items (natural vs fake, human-like vs machine-like, conscious vs unconscious, lifelike vs artificial, and adaptive vs rigid) using a 7-point Likert scale based on a horizontal visual analog scale. The scores on the scale were summed and averaged to create a mean composite score. A higher score indicates more human-like HeartBot messages. The scale was developed based on a previous study [24]. The internal consistency of the scale was strong with Cronbach α=0.90 in our study sample, indicating a high level of internal consistency.

##### Message Effectiveness

In the AI chatbot behavior change model [8], message effectiveness is classified under “user experiences” in domain 4, evaluating mechanisms and outcomes, assessing the perceived usefulness and convenience of chatbot interactions. Participants rated the self-reported effectiveness of chatbot messages using the “effectiveness scale” in the postintervention survey. The scale was originally developed based on previous literature [25,26]. The scale consists of 5 items (effective vs ineffective, helpful vs unhelpful, beneficial vs not beneficial, adequate vs not adequate, and supportive vs not supportive) using a 7-point Likert scale based on a horizontal visual analog scale. The scores on the scale were summed and averaged to create a mean composite score. A higher score indicates greater message effectiveness of HeartBot. The internal consistency of the scale was strong, as evidenced by Cronbach α=0.93 in our study sample.

##### Attitude Toward AI

To investigate the attitude toward AI chatbots, participants were asked the following question on the postintervention survey: “*How positive or negative do you feel about the use of artificial intelligence in healthcare?*” There are 5 response options—very positive, positive, neutral, negative, and very negative.

##### Perception of Chatbot Identity (Human vs AI Chatbot)

To determine the perception of the identity of HeartBot, participants were asked the following question at the postintervention survey: “*Do you think you texted a human or an artificial intelligent chatbot during your conversation?*” There were 2 response options—human or artificial agent.

### Statistical Analysis

Descriptive analyses were used to describe participants’ sociodemographic backgrounds, cardiovascular risks, medication, and AI chatbot interactions and evaluations. The sample was split based on the perception of chatbot identity as a human versus an artificial agent. Chi-square test, Fisher exact test, and Wilcoxon rank-sum test were used to compare the differences in baseline sample characteristics of the 2 subsamples.

Race or ethnicity and education were recoded into dichotomous variables: non-White or White and “completed college or graduate school” or “less than high school or did not complete college,” respectively, in a logistic regression analysis. Attitude toward the AI chatbot was divided into 3 categories: positive, neutral, and negative. Recoding several variables was aimed at improving statistical power.

Additionally, univariate logistic regression analyses were performed to estimate the relationships between the dependent variable (ie, the perception of chatbot identity) and each independent variable with sociodemographic factors, previous AI chatbot use, AI chatbot interaction, and AI chatbot evaluation. The logistic regression analyses calculated the point estimate and 95% CI of the odds ratio (OR), which is associated with the perception of the chatbot identity as being a human. In the logistic regression analyses, if the 95% CI of the OR includes 1.0, there is no statistically significant relationship between the independent and dependent variables.

Finally, a multivariable logistic regression analysis was conducted to determine factors that were associated with participants’ perception of the chatbot identity as being a human. The final multivariable regression model includes age, race or ethnicity, education, previous AI chatbot use, conversation lengths with HeartBot, message humanness, message effectiveness, and attitude toward AI. The independent variables ensured face validity (ie, age, race, and education), and the potential confounders referred to in literature [22] were entered into a multivariate regression model. The other potential confounding factors were selected from the AI chatbot behavior change model [8], including previous AI chatbot use, conversation lengths with HeartBot, message humanness, message effectiveness, and attitude toward AI. This model guided our selection of covariates to better understand how participants evaluated the interaction and how specific communication features may have affected their experience. Multicollinearity was tested to ensure that independent variables were not highly correlated. The variance inflation factor values of all independent variables ranged from 1.13 to 2.12 (mean 1.47; SD 0.83), indicating an acceptable range and no multicollinearity in the variables. Statistical significance was set at a 2-sided *P* value <.05. All analyses were performed using Stata (version.18.0; StataCorp) [27].

### Ethical Considerations

This study adhered to the ethical principles outlined in the Declaration of Helsinki and received approval from the University of California, San Francisco Institutional Review Board (approval 23‐39793). Written informed consent was obtained from all participants before enrollment. Participation was voluntary, and participants could withdraw at any time without penalty. All data were deidentified before analysis and stored on secure, password-protected servers accessible only to the research team. Participants who completed all study procedures received a US $20 Amazon electronic-gift card as compensation.

## Results

### Sample Characteristics

Multimedia Appendix 3 presents screening, enrollment, and follow-up of the study participants. A total of 92 participants completed the baseline, HeartBot interaction, and postintervention surveys (Table 1). The mean age of participants was 45.9 (SD 11.9, range 26-70) years. In total, 40% (n=37) of participants identified their race and ethnicity as White or Caucasian, 24% (n=22) as Black or African American, and 21% (n=19) as Hispanic or Latino Americans. Furthermore, 72% (n=66) reported completing college or graduate school. Of the total, 45% (n=41) of participants reported experiencing menopause; 36% (n=33) of participants reported their BMI was 30 or above; and 27% (n=25) reported taking blood pressure medication. In addition, 58% (n=53) reported experiencing a previous interaction with an AI chatbot. The most popular types of chatbots were ChatGPT (OpenAI; n=22, 24%) and Siri (Apple Inc; n=20, 22%).

**Table 1:** Sample characteristics in respondent perception of chatbot identity as a human versus an artificial agent (N=92).

Characteristics					Overall (N=92)			Human (n=31)			Artificial agent (n=61)			*P* value^a^
**Sociodemographic factors**														
	Age (y), mean (SD; range)			45.9 (11.9; 26-70)			46.3 (12.2; 28-70)			45.6 (11.9; 26-68)			.82	
	**Race or ethnicity, n (%)**												.41	
		American Indian or Alaskan Native	1 (1.1)			0 (0)			1 (1.6)					
		Asian	6 (6.5)			4 (12.9)			2 (3.3)					
		Black or African American	22 (23.9)			9 (29)			13 (21.3)					
		Hispanic or Latino	19 (20.7)			4 (12.9)			15 (24.6)					
		Native Hawaiian or Other Pacific Islander	2 (2.2)			0 (0)			2 (3.3)					
		White or Caucasian	37 (40.2)			12 (38.7)			25 (41)					
		More than 1 race or ethnicity	5 (5.4)			2 (6.5)			3 (4.9)					
	**Education, n (%)**												.27	
		No more than high school or did not complete college	26 (28.3)			11 (35.5)			15 (24.6)					
		Completed college or graduate school	66 (71.7)			20 (64.5)			46 (75.4)					
	**Household income, n (%)**												.16	
		Less than $75,000, do not know, or decline to respond	33 (57.6)			21 (67.7)			32 (52.5)					
		$75,000 or above	39 (42.4)			10 (32.3)			29 (47.5)					
	**Marital status, n (%)**												.76	
		Never married	21 (22.8)			8 (25.8)			13 (21.3)					
		Currently married or cohabitating	59 (64.1)			20 (64.5)			39 (63.9)					
		Divorced or widowed	12 (13)			3 (9.7)			9 (14.8)					
	**Employment status, n (%)**												.77	
		Employed full-time or part-time	56 (60.9)			18 (58.1)			38 (62.3)					
		Unemployed or looking for a job, student, or homemaker	17 (18.5)			7 (22.6)			10 (16.4)					
		Retired, disabled, or other	19 (20.7)			6 (19.4)			13 (21.3)					
	Immigration experience to the United States, n (%)			12 (13)			5 (16.1)			7 (11.5)			.37	
**Cardiovascular risk factors or medication intake**														
	Menopause, n (%)			41 (44.6)			14 (45.2)			27 (44.3)			.94	
	**BMI (kg/m** ^**2**^ **), n (%)**												.084	
		Less than 30	58 (63.7)			16 (51.6)			42 (70)					
		30 or above	33 (36.3)			15 (48.4)			18 (30)					
	Smoking in the past 30 days, n (%)			14 (15.2)			4 (12.9)			10 (16.4)			.46	
	Physical activity ≥150 min per week, n (%)			56 (60.9)			20 (64.5)			36 (59)			.61	
	Family history of heart disease, n (%)			13 (14.1)			4 (12.9)			9 (14.8)			.54	
	Blood pressure medication, n (%)			25 (27.2)			6 (19.4)			19 (31.2)			.23	
	Cholesterol medication, n (%)			16 (17.4)			5 (16.1)			11 (18)			.82	
	Diabetes medication, n (%)			17 (18.5)			6 (19.4)			11 (18)			.88	
	Taking aspirin daily, n (%)			13 (14.1)			5 (16.1)			8 (13.1)			.46	
**HeartBot interaction**														
	Previous AI^b^ chatbot use, n (%)			53 (57.6)			17 (54.8)			36 (59)			.70	
	Conversation length (words), mean (SD; range)			82.5 (61.9; 34-377)			81.8 (67.0; 36-360)			82.8 (59.8; 34-377)			.18	
	Conversation length (minutes), mean (SD; range)			13.0 (7.8; 5.6-42.2)			13.1 (9.6; 5.6-42.2)			12.9 (6.8; 5.6-40.3)			.33	
	Number of questions asked to HeartBot (at least one), n (%)			27 (29.4)			7 (22.6)			20 (32.8)			.31	
**HeartBot evaluation**														
	Message humanness, mean (SD; range)			5.2 (1.2; 2.0-7.0)			5.7 (1.1; 3.4-7.0)			4.9 (1.2; 2.0-7.0)			.003	
	Message effectiveness, mean (SD; range)			5.7 (1.2; 1.0-7.0)			5.9 (0.9; 3.4-7.0)			5.6 (1.4; 1.0-7.0)			.62	
	**Attitude toward AI, n (%)**												≥.99	
		Positive	35 (38)			12 (38.7)			23 (37.7)					
		Neutral	44 (47.8)			15 (48.4)			29 (47.5)					
		Negative	13 (14.1)			4 (12.9)			9 (14.8)					

^a^*P* value was calculated by chi-square test, Fisher exact test, or Wilcoxon rank-sum test.

^b^AI: artificial intelligence.

### HeartBot Interaction

As illustrated in Table 1, while 34% (n=31) of participants identified the chatbot as a human, 66% (n=61) of participants reported they interacted with an artificial agent. The mean (SD, range) and median (IQR) of conversation length with HeartBot by word count and minute were 82.5 (SD 61.9, range 34-377), 64.5 (IQR 46.0-49.0) words and 13.0 (SD 7.8, range 5.6-42.2), 10.6 (IQR 8.5-13.9) minutes, respectively. The mean scores of message humanness and message effectiveness were 5.2 (SD 1.2, range 2.0-7.0) and 5.7 (SD 1.2, range 1.0-7.0), respectively. Furthermore, 38% (n=35) of participants had a positive feeling for AI. In the bivariate analysis, the mean score of message humanness was significantly higher in the group who answered the chatbot identity as a human compared with the group who thought they were interacting with an artificial agent (*P*=.003).

Table 2 presents the unadjusted and adjusted ORs from multivariable logistic regression analysis results for predicting the perception of chatbot identity as a human versus an artificial agent. In the unadjusted model, the score of message humanness was significantly associated with the perception of chatbot identity as a human compared with an artificial agent (unadjusted OR 1.81, 95% CI 1.19-2.77; *P*=.006). In the adjusted model, only the score of message humanness was significantly associated with the perception of chatbot identity as a human compared with an artificial agent (adjusted OR 2.37, 95% CI 1.26-4.48; *P*=.007), controlling for age, race or ethnicity, education, previous AI chatbot use, conversation length with HeartBot, message effectiveness, and attitude toward AI.

**Table 2:** Unadjusted and adjusted odds ratios from multivariable logistic regression analysis for predicting the perception of chatbot identity as being a human (N=92).

Variables			OR^a^ (95% CI)		*P* value		AOR^b^ (95% CI)		*P* value
Age			1.01 (0.97-1.04)		.79		0.99 (0.95-1.04)		.80
**Race and ethnicity**									
	Non-Hispanic White	1 (Reference)		—^c^		1 (Reference)		—	
	Non-White^d^	1.10 (0.45-2.66)		.83		1.15 (0.37-3.57)		.81	
**Education**									
	Less than high school or did not complete college	1 (Reference)		—		1 (Reference)		—	
	Completed college or graduate school	0.59 (0.23-1.52)		.28		0.56 (0.19-1.66)		.29	
**Previous AI^e^** **chatbot use**									
	No	1 (Reference)		—		1 (Reference)		—	
	Yes	0.84 (0.35-2.02)		.70		0.93 (0.31-2.79)		.90	
Conversation length (words)			1.00 (0.99-1.01)		.94		1.00 (0.99-1.01)		.93
Message humanness			1.81 (1.19-2.77)		.006		2.37 (1.26-4.48)		.007
Message effectiveness			1.23 (0.84-1.81)		.29		0.70 (0.37-1.33)		.28
**Attitude toward AI**									
	Negative	1 (Reference)		—		1 (Reference)		—	
	Neutral	1.16 (0.31-4.41)		.82		1.16 (0.22-6.07)		.87	
	Positive	1.17 (0.30-4.62)		.82		1.01 (0.16-6.43)		.99	

^a^OR: odds ratio.

^b^AOR: adjusted odds ratio.

^c^Not applicable.

^d^Non-White included American Indian, Alaskan Native, Asian, Black or African American, Hispanic or Latino, Native Hawaiian, other Pacific Islander, and multiracial individuals.

^e^AI: artificial intelligence.

## Discussion

### Principal Findings

This study explored whether and why people attribute different identities to an AI chatbot, specifically examining the extent to which AI chatbots are perceived as a human versus an artificial agent among women, and identified key factors influencing these perceptions. A key finding in this study was that women who perceived a higher degree of message humanness were more likely to identify the chatbot (HeartBot) as human. In contrast, neither the message effectiveness nor general attitudes toward AI influenced the perception of the chatbot identity. These results suggest that human-like chatbot communication is critical in shaping users’ perceptions of chatbot identity. However, in this secondary data analysis with a limited sample size (N=92), we were unable to sufficiently adjust for cultural, demographic, or contextual characteristics. Thus, caution needs to be exercised when interpreting message humanness in relation to identifying the chatbot as human.

This study’s findings are consistent with existing research findings. According to Go and Sundar [28], 3 factors that influence humanness among AI chatbots are visual cues (eg, using human figures), conversational cues (eg, interactive or contingent messages), and identity cues (eg, human-like names or identities). Conversational cues refer to human-like contingent conversational markers that increase expectations for human-like communication with chatbots, such as using empathetic phrases, polite statements, and acknowledging users’ previous responses [29]. Such conversational cues can enhance the humanness of conversations delivered by chatbots. Assessing the characteristics of conversational cues and perceived humanness in conversations with AI chatbots is important because how users perceive a chatbot’s identity can have implications on their expectations and evaluations of the chatbot’s performance and effectiveness. If users assume the chatbot identity as an artificial agent, they are more likely to assess the quality of chatbot performance based on their existing stereotypes of chatbots [12,28,29]. In contrast, if they assume a chatbot is human, they are more likely to assess the quality of chatbot performance based on their expectations of other humans. Typically, when users perceive a chatbot as more human-like, they expect better and more natural performance from the chatbot than when they perceive it as an AI agent [12,28,29].

Subjective expectations of the chatbot’s performance matter, and if these expectations are not met, user evaluations of the chatbot will be poorer. This is explained by the “expectancy violation effect” [30]. Thus, calibrating users’ expectations of AI chatbots is an important consideration in designing the characteristics and conversational features of chatbots. For instance, past research has shown that message contingency, defined as human-like continuous dialogues remembering previous responses, could enhance a chatbot’s social presence, which further increased users’ perceived intelligence and friendliness of the chatbot [28]. In our study, even though all participants were informed that they were interacting with a chatbot named “HeartBot,” a significant portion of the women still thought they were interacting with a human. This “misperception” could be due to the fact that they highly evaluated the chatbot messages’ naturalness and humanness.

Interestingly, the message effectiveness and attitude toward the HeartBot conversations were not significantly associated with the perception of chatbot identity in this study. A previous study similarly revealed that the actual performance level of a chatbot did not influence its perception as a human [31]. Our findings indicate that perceiving an AI chatbot as a human or an artificial agent is largely dependent on the encoding and decoding of actual conversational messages, rather than the conversational context or the impact of the conversation.

While previous research studies have shown the importance of anthropomorphic cues in chatbot perception [28,29], little is known about how these perceptions play out in health care contexts, where the stakes are not just user satisfaction or technology adoption, but also patient trust in the information, readiness to change, willingness to follow AI-generated health advice, and adherence to recommendations. We highlight that this study offers a novel health care–focused theoretical insight by showing that even when message effectiveness is held constant, message humanness (indicating relational or human-like qualities in chatbot communication) significantly shapes how participants perceive the chatbot identity, which can, in turn, influence their openness to engaging with and trusting digital health tools.

While the chatbot introduced itself as HeartBot at the beginning of the conversation, approximately 1 in 3 participants incorrectly perceived that they were interacting with a person. Since the univariate and multivariate analyses showed the nonsignificant relationships between sociodemographic factors and the perception of the chatbot identity, the perceived message humanness level may influence the perception of the chatbot identity regardless of differences in sociodemographic factors among women.

Considering ethical concerns for AI chatbot applications in the health care field, this study highlights that system designers and researchers need to recognize that some users may perceive they are interacting with a person, while others may find it clear that they are interacting with an AI chatbot. This misperception can lead users to develop unrealistic expectations of the chatbot’s capabilities, potentially impacting informed consent and clinical decision-making. While earlier research showed that using human identity or strategically hiding AI chatbots’ identity may be advantageous in enhancing the user experience [32-34], we argue it is no longer ethical and can be counterproductive in forming the right expectations and useful interactions with AI chatbots. In this study, the chatbot introduced itself as HeartBot at the beginning of the conversation. However, the findings of this study suggest that this alone may be insufficient to prevent the misperception of the chatbot identity as a human. Therefore, we recommend that future research in health chatbot design need to prioritize explicit and repeated chatbot identity disclosure, with clear communication of the chatbot’s capabilities, limitations, and data use policies before user interaction. These practices would protect user autonomy and clinical decision-making processes. Given the limited number of health intervention studies that examine how identity disclosure affects trust with an AI chatbot, further investigation in this area is warranted.

Other ethical concerns when designing human-like chatbots in health care contexts include overtrust in an AI chatbot, fairness and bias, and accountability. First, a highly empathetic or responsive chatbot might foster overtrust with an AI chatbot, leading users to rely on it instead of seeking professional health support. This could compromise patient safety and delay appropriate care because AI algorithms sometimes make inaccurate clinical recommendations and provide inaccurate health information [35]. To avoid these issues, it is necessary for system developers and researchers to inform users of the boundaries of what the AI chatbot can and cannot do. Second, AI systems can be biased, which may perpetuate existing health disparities, particularly when interacting with marginalized groups. For example, an AI algorithm based on research from predominantly White participants may discriminate against racially and culturally minority communities or lead to inaccurate provision of information [35,36]. Assessing the potential bias in the dataset or model design, and incorporating inclusive designs with diverse user input, are essential to mitigate bias. Third, when an AI chatbot gives incorrect or harmful health advice, it is unclear who is responsible for the chatbot’s outputs. Lack of accountability may undermine trust in an AI chatbot and user safety. If a recommendation made by a clinical decision supporting AI chatbot leads to a negative outcome for users, it is unclear who to assign the responsibility to or to prevent it from happening again. Clear definitions of accountability and AI systems for feedback and redress when misinformation occurs are needed to enhance users’ trustworthiness with an AI chatbot and to prevent poor patient outcomes. In summary, addressing overtrust, bias, and accountability is crucial when designing human-like AI chatbots to ensure user safety and trust.

The findings of this study offer valuable clinical implications for designing human-like AI chatbots that can support innovative health interventions, including chronic disease management, symptom monitoring, counseling, and health education. For example, an AI chatbot with human-like features that monitors patient data and provides personalized lifestyle recommendations may enhance patient trust and motivation, thereby improving retention and adherence to preventive interventions for chronic diseases. Given the limited empirical evidence that directly tested the relationship between human-like chatbot characteristics and health outcomes, further investigations are needed to clarify these relationships and optimize chatbot designs for health interventions.

### Limitations

It is important to acknowledge several limitations when interpreting the study’s findings. First, the convenience sampling method may have affected the study findings due to selection bias. Second, only female adults in the United States were included in the HeartBot program, which may limit the generalizability of the findings to male adults and populations in other countries. Third, we could not conduct the subgroup analysis due to the limited sample size (N=92). Furthermore, since the sample size was limited, the findings should be interpreted with caution and considered hypothesis-generating rather than confirmatory. Finally, the anthropomorphism scale and the effectiveness scale are self-report instruments. While the Cronbach α showed high internal consistency in both scales (Cronbach α=0.90 and 0.93, respectively), it may not objectively reflect the conversation quality with HeartBot. These self-reported measures may lead to overestimating the level of message humanness or message effectiveness. Future studies are needed to combine subjective and objective measures to evaluate the conversation quality of the human-chatbot interactions.

### Conclusion

This study highlights the significant role that perceived message humanness plays in shaping the user’s perception of chatbot identity. Conversely, message effectiveness and attitudes toward AI did not significantly influence the perception of the chatbot identity as being a human. Findings suggest that the perceived human-like attributes primarily drive users to attribute a human identity to the chatbot, specifically in health care settings where user trust and engagement are crucial. This study provides a theoretical foundation for understanding human-AI chatbot interactions and offers practical insights for designing person-centered AI chatbots in health care. Further research is needed to explore the relationship between message humanness, chatbot identity, and health outcomes to optimize the design of AI chatbots in the health-related fields.

## Appendix

Multimedia Appendix 1 STROBE Checklist.

Multimedia Appendix 2 The artificial intelligence chatbot behavior change model.

Multimedia Appendix 3 Flow diagrams: screening, enrollment, and follow-up of the study participants.

## Abbreviations

AI: artificial intelligence
OR: odds ratio
RCT: randomized controlled trial
REDCap: Research Electronic Data Capture
STROBE: Strengthening the Reporting of Observational studies in Epidemiology

## Data Availability

The datasets generated or analyzed during this study are available from the corresponding author on reasonable request.
