# Supplementary material for: Message Humanness as a Predictor of AI’s Perception as Human: Secondary Data Analysis of the HeartBot Study
Source: JMIR AI. 2026 Feb 3;5:e67717. doi: 10.2196/67717 (PMC12914229; doi:10.2196/67717)
Supplement: Multimedia Appendix 2 [file ai_v5i1e67717_app2.docx]

[View full-text article in PMC](https://pmc.ncbi.nlm.nih.gov/articles/PMC7557439/)

J Med Internet Res

. 2020 Sep 30;22(9):e22845. doi: [10.2196/22845](https://doi.org/10.2196/22845)

- Copyright and License information

©Jingwen Zhang, Yoo Jung Oh, Patrick Lange, Zhou Yu, Yoshimi Fukuoka. Originally published in the Journal of Medical Internet Research (http://www.jmir.org), 30.09.2020.

This is an open-access article distributed under the terms of the Creative Commons Attribution License (<https://creativecommons.org/licenses/by/4.0/>), which permits unrestricted use, distribution, and reproduction in any medium, provided the original work, first published in the Journal of Medical Internet Research, is properly cited. The complete bibliographic information, a link to the original publication on <http://www.jmir.org/>, as well as this copyright and license information must be included.

[PMC Copyright notice](https://pmc.ncbi.nlm.nih.gov/about/copyright/)

**Figure 1.**

https://support.jmir.org/hc/en-us/articles/37982552280987-Submitting-Your-Manuscript-to-JMIR-Publications-A-Guide-for-Authors?__hsfp=1774233285&__hssc=102212634.1.1758834957032&__hstc=102212634.b67dd9f6a4c17f37a854d483018d972f.1758834957031.1758834957031.1758834957031.1#h_01JXNF8F7NG5Q2RQ6A3F1406VA


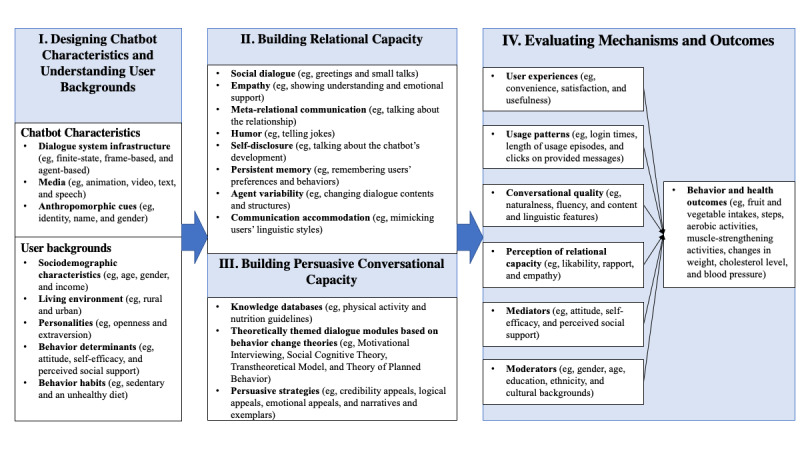


The artificial intelligence chatbot behavior change model.
