# Supplementary material for: Message Humanness as a Predictor of AI’s Perception as Human: Secondary Data Analysis of the HeartBot Study
Source: JMIR AI. 2026 Feb 3;5:e67717. doi: 10.2196/67717 (PMC12914229; doi:10.2196/67717)

## **Multimedia Appendix 3**.

Figure S1. Flow diagrams: screening, enrollment, and follow-up of the study participants.


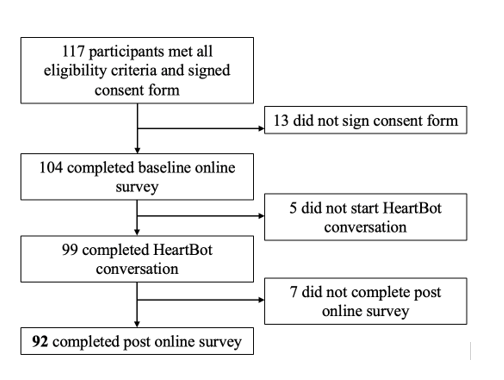

Supplement: Multimedia Appendix 3 [file ai_v5i1e67717_app3.docx]
